# Supplementary material for: Molecular Recognition of Imidazole-Based Drug Molecules by Cobalt(III)- and Zinc(II)-Coproporphyrins in Aqueous Media
Source: Molecules. 2023 Jan 18;28(3):964. doi: 10.3390/molecules28030964 (PMC9920418; doi:10.3390/molecules28030964)
Supplement: Supplementary file 1 [file molecules-28-00964-s001.zip › molecules-2149206 Supplementary materials .pdf]

molecules

Supplementary materials of the article

Molecular Recognition of Imidazole-Based Drug Molecules by Cobalt(III)- and Zinc(II)-Coproporphyrins in Aqueous Media

Galina Mamardashvili, Elena Kaigorodova, Ivan Lebedev, Nugzar Mamardashvili *

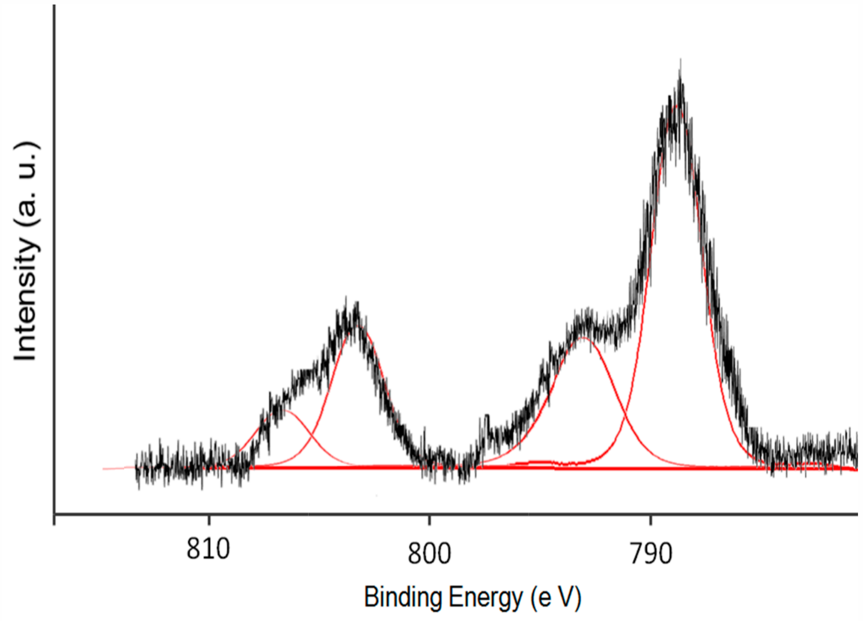

Figure.S1. Deconvoluted Co 2p XPS spectrum of the $CoCP(Im)_2$ powder samples.

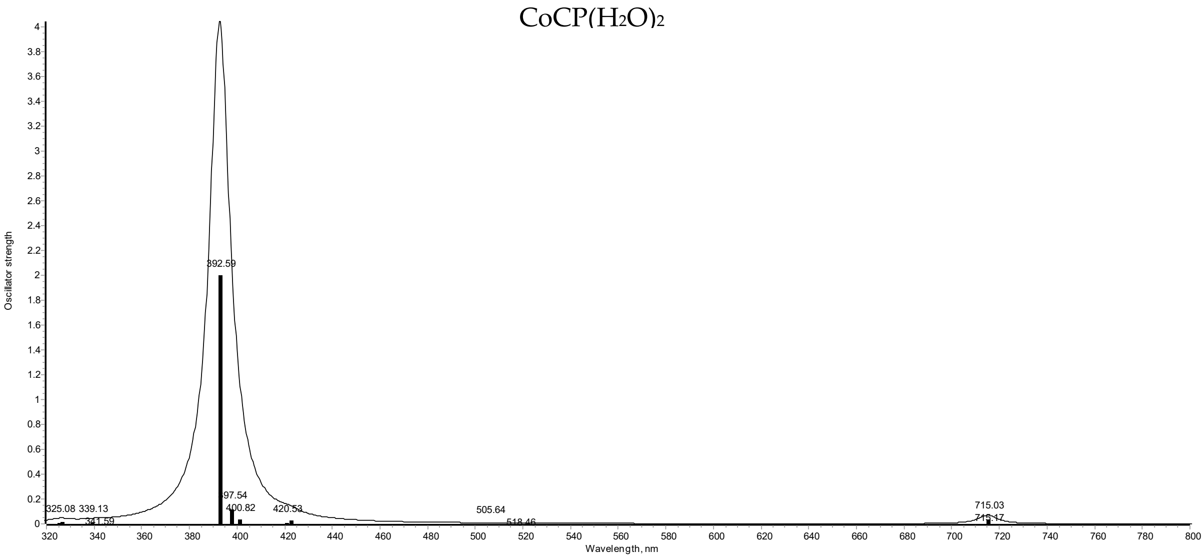
CoCP(H2O)2
Oscillator strength
Wavelength, nm
392.59
397.54
400.82
420.53
325.08
339.13
341.59
505.64
518.46
715.03
715.17

(a)

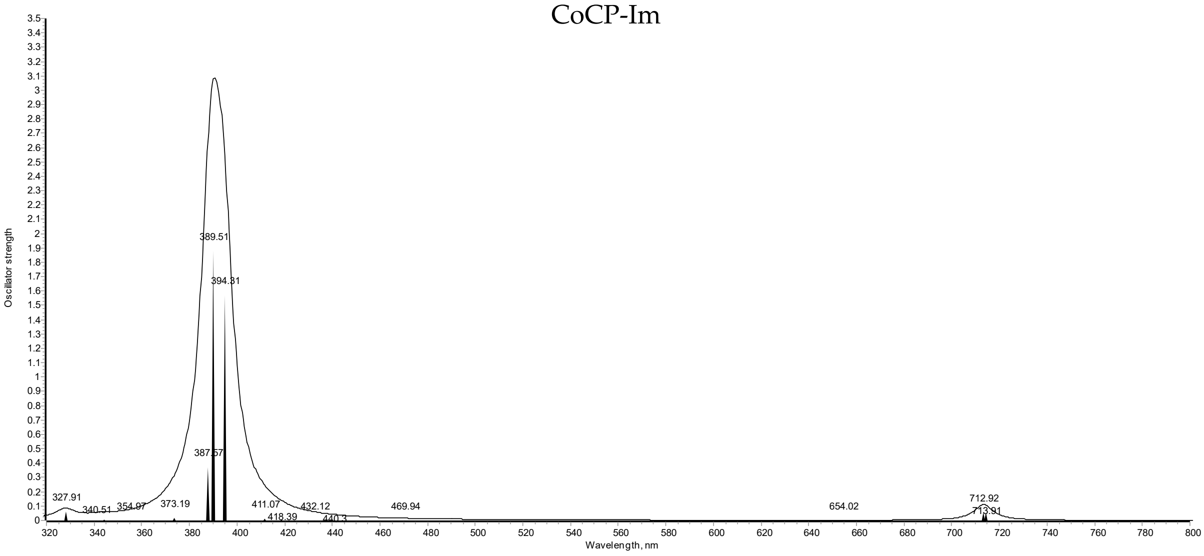
CoCP-Im
Oscillator strength
Wavelength, nm
389.51
394.31
387.57
327.91
340.51
354.97
373.19
411.07
418.39
432.12
440.3
469.94
654.02
712.92
713.91

(b)

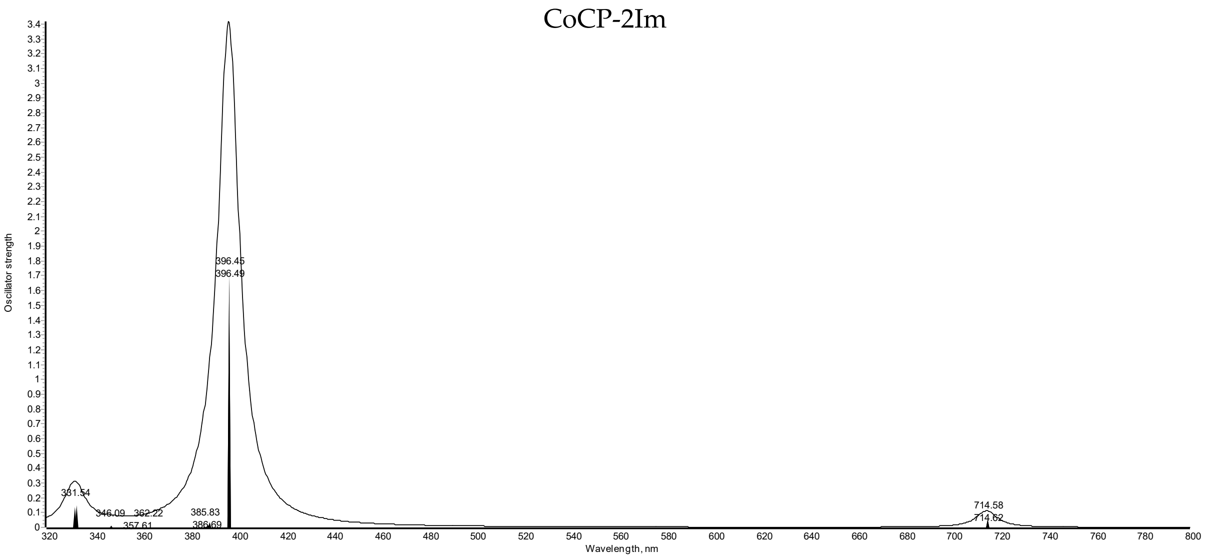

(c)

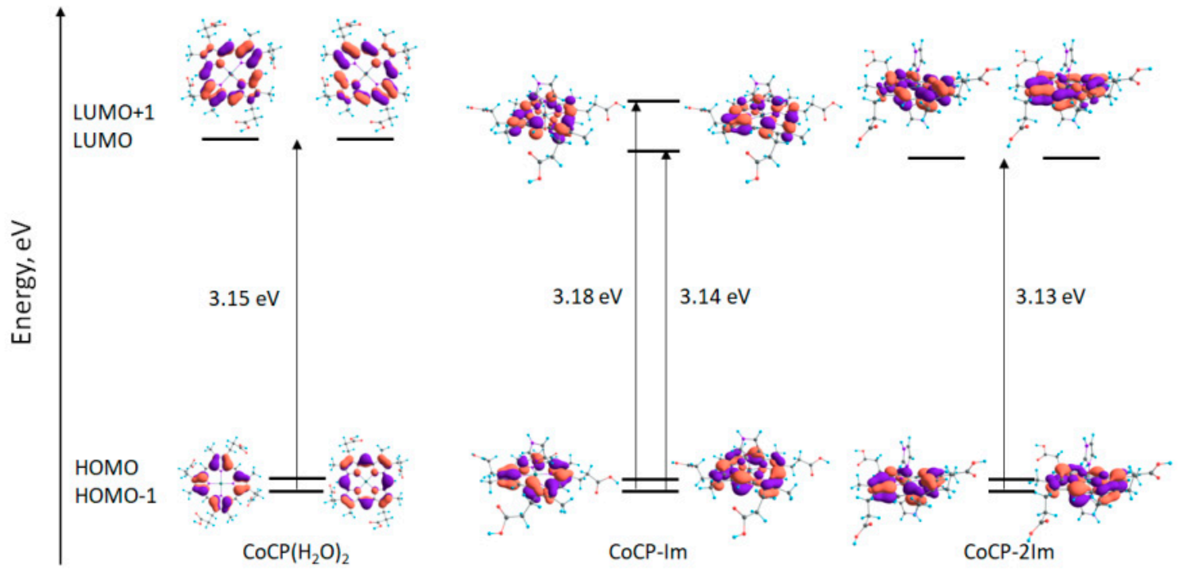

(d)

Figure S2. TDDFT absorption spectra of CoCP (a), CoCP-(Im) (b) and CoCP-$(Im)_2$ (c), Energy and type of calculated frontier molecular orbitals of CoCP, CoCP(Im) and CoCP$(Im)_2$ (d)

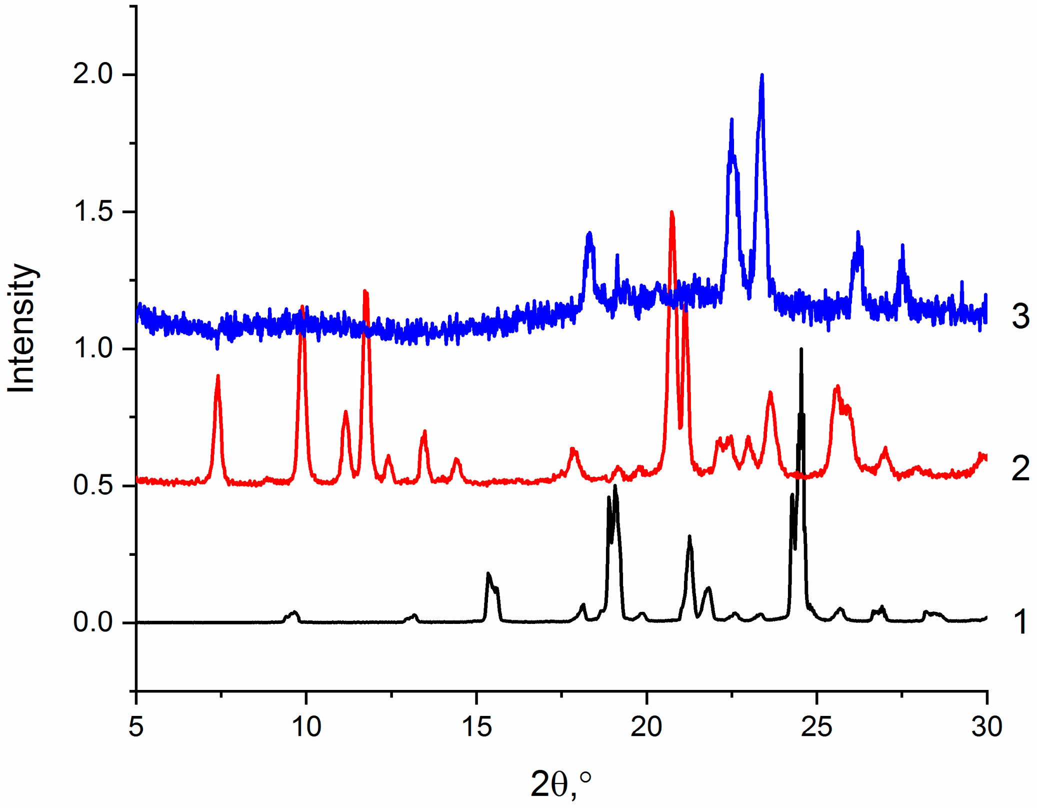

Figures S3. Powder XRD profiles of 1 (L2), 2(CoCP), 3 [CoCP(L2)$_2$]
